# Insights into the Regulatory Role of MicroRNAs in *Penaeus monodon* Under Moderately Low Salinity Stress

**DOI:** 10.3390/biology14040440

**Published:** 2025-04-18

**Authors:** Jianzhi Shi, Song Jiang, Yangyang Ding, Hongshan Diao, Wenzhe Li, Yundong Li, Jianhua Huang, Lishi Yang, Qibin Yang, Falin Zhou

**Affiliations:** 1 Key Laboratory of Efficient Utilization and Processing of Marine Fishery Resources of Hainan Province, Sanya Tropical Fisheries Research Institute, Sanya 572018, China; shijianzhi1989@163.com; 2 Key Laboratory of South China Sea Fishery Resources Exploitation and Utilization, Ministry of Agriculture and Rural Affairs, South China Sea Fisheries Research Institute, Chinese Academy of Fishery Sciences, Guangzhou 510300, China; tojiangsong@163.com (S.J.); dingyangyang93@163.com (Y.D.); 17875808801@163.com (H.D.); 15836677553@163.com (W.L.); liyd2019@163.com (Y.L.); huangjianhua@scsfri.ac.cn (J.H.); yangls2016@163.com (L.Y.); 3 Shenzhen Base of South China Sea Fisheries Research Institute, Chinese Academy of Fishery Sciences, Shenzhen 518108, China

**Keywords:** microRNA, *Peaneus monodon*, low salinity, regulatory roles

## Abstract

MicroRNAs (miRNAs) play crucial roles in regulating various biological processes in crustaceans, including stress responses. Under acute low salinity stress conditions, miRNAs exhibit dynamic expression patterns that significantly influence the physiological and molecular responses of the shrimp. However, research on miRNAs in *P. monodon* is very limited, and their functions under low salinity stress remain unclear. In this study, by using high-throughput sequencing technology, we identified miRNAs and investigated their regulatory mechanism in *P. monodon* under low salinity stress. A total of 118 miRNAs were differentially expressed after low salinity exposure. These miRNAs were found to target genes involved in metabolism, pathogen infection, immune response and stress signaling pathways. By modulating the expression of these target genes, miRNAs were able to fine-tune the stress response of *P. monodon*, thereby enhancing its tolerance to low salinity. This study provides new insights into the regulatory roles of miRNAs in the stress response of aquatic organisms and suggests potential targets for genetic improvement to enhance stress tolerance in *P. monodon* aquaculture.

## Simple Summary

This study investigates how microRNAs (miRNAs) help black tiger shrimp (*Penaeus monodon*) adapt to moderately low salinity stress (17 ppt), a common challenge in aquaculture due to climate change. Using high-throughput sequencing, researchers identified 118 miRNAs in shrimp hepatopancreas that showed significant changes in expression after salinity exposure. These miRNAs regulate genes involved in metabolism, immune response, and stress adaptation. Key findings revealed that low salinity disrupts growth-related processes (e.g., digestion, hormone secretion) and increases disease susceptibility, while miRNAs help coordinate stress responses by modulating pathways like immune signaling and nervous system function. The results provide new insights into shrimp stress tolerance mechanisms and highlight potential molecular targets for breeding more resilient shrimp varieties, supporting sustainable aquaculture practices.

## 1. Introduction

The black tiger shrimp (*Penaeus monodon*) is a tropical shrimp commonly found in the Indo-Pacific region. With the rapid growth in global demand, *P. monodon* has become an economically important shrimp species [1]. In 2023, the production of *P. monodon* reached 128,000 tons, making it the second largest marine shrimp species cultured in China [2]. However, extreme climate changes, such as rising temperatures and increased occurrence of heavy rainfall, are currently impacting the culture environment. The rise of water temperature alters the growth and feed conversion of cultured shrimp, leading to high mortality rates [3]. Similarly, abnormal rainfall causes sudden shifts in salinity, which can affect the dynamics of microorganisms in water and the susceptibility of shrimp to pathogen infection [4]. Environmental stress has become a significant threat to the quantity and quality of shrimp aquaculture by affecting metabolic processes or increasing the risk of disease outbreaks.

As a euryhaline crustacean species, *P. monodon* can survive in a wide range of salinities [5]. This marine species has been cultured in low salinity groundwater in South China. However, it has been observed that low salinity significantly depresses the growth performance of shrimp [6]. This is primarily because, in order to maintain osmotic balance at low salinity, shrimp are forced to expend more energy for osmoregulation. Additionally, inland low salinity waters are deficient in K^+^ and Mg^2+^, which are crucial for the growth and survival of shrimp [7]. On the other hand, low salinity could increase the toxicity of nitrite (NO_2_^−^), nitrate (NO_3_^−^) and ammonia (NH_3_), leading to environmental stress for shrimp [8]. Thus, further exploration is needed to understand the physiological adaptations of osmoregulation in shrimp, and a practical way to improve growth performance in *P. monodon* at low salinity needs to be identified.

The regulatory mechanisms of penaeid shrimp under low salinity conditions have garnered significant scientific interest. In *Litopenaeus vannamei*, previous studies on salinity adaptation mainly focused on the function of ion transporters and ion channels in osmoregulation [9,10]. Several studies have used omic approaches to reveal the genes and pathways that respond to salinity change in different tissues. Pathways related to signal transduction, oxidative, lipid and fatty acid metabolism were activated after low salinity stress [11,12,13]. Moreover, two vital genes—mitogen-activated protein kinase kinase and cold shock domain binding protein—involved in stress resistance and immunity were identified as regulators of low salinity in *P. monodon* [14,15]. Although our understanding of salinity adaptation in shrimp is increasing, information about the roles of microRNA (miRNA) in improving shrimp osmoregulation capacity remain limited.

MicroRNAs belong to a class of small non-coding RNAs, approximately 22 nucleotides in length, that regulate the post-transcription expression of messenger RNAs through sequence-specific base pairing and the consequent silencing of target mRNAs [16]. The miRNAs are involved in many biological processes, such as cellular differentiation, proliferation, apoptosis and immunity [17,18,19,20]. With the development of high-throughput sequencing technologies, there has been a rapid growth in the number of identified miRNAs in aquatic animals. Recently, several studies have demonstrated the role of miRNAs in the stress response of shrimps [21,22,23]. In a previous study, we also identified miRNAs in *P. monodon* under severe low-salinity stress (3 ppt). A total of 43 differentially expressed miRNAs were selected as putative key modulators, whose target genes formed a network to maintain homeostasis [24]. However, another dimension of salinity variation requires attention. Under the influence of climate change, frequent summer rainstorms in South China have led to a significant reduction in seawater salinity, decreasing from ~30 ppt to below 20 ppt, resulting in moderately low salinity stress on shrimp. It has been proven that the response of *P. monodon* varies with salinity levels. Extremely low salinity (<2.5 ppt) induced the lowest survival and growth rate, while moderately low salinity (10 ppt) had a relatively small impact [6]. Additionally, a difference in rearing salinity could also affect the microbiota and gene expressions of shrimp intestines [25]. Thus, to gain deeper insights into the regulatory role of microRNAs in *P. monodon*, further experiments related to moderately low salinity are needed.

In crustaceans, the hepatopancreas acts as a multifunctional organ, coordinating nutrient absorption and digestion, energy storage, lipid and carbohydrate metabolism, detoxification, and immunity [26]. Transcriptome analysis has been widely employed to investigate gene expression patterns in shrimp under low-salinity conditions [27,28]. However, the regulatory roles of miRNAs in the low-salinity adaptation of *P. monodon* remain poorly understood. In the present study, we constructed small RNA libraries from the hepatopancreas of black tiger shrimp at four time points after moderately low salinity stress (0, 6, 24, and 96 h). Based on differentially expressed miRNAs and their target genes, the molecular mechanisms under moderately low salinity stress were comprehensively analyzed. Our results will expand the understanding of shrimp miRNA regulation and provide a scientific basis for the breeding of improved varieties of *P. monodon*.

## 2. Materials and Methods

### 2.1. Animals Used for the Experiments

*P. monodon* for the experiments were acquired from the Shenzhen experimental base of the South China Sea Fisheries Research Institute, Chinese Academy of Fisheries Sciences, Guangzhou, China. Shrimp weighing 10 ± 2 g were cultivated in aerated and filtered seawater maintained at a temperature of 28–30 °C and a salinity level of 30–32 ppt, while being fed three times a day with commercial feed (Dongteng Feed, Zhanjiang, China). Approximately one third of the water in each tank was replaced every day.

### 2.2. Treatment of Low Salinity Stress

Following previous works, a salinity level of 17 ppt and a duration of 96 h were chosen to induce a moderately low salinity stress treatment [14,15]. Experimental shrimp were randomly selected and exposed to salinity 17. Three plastic tanks were set up, with each tank containing 30 shrimp. Hepatopancreas tissues of randomly selected individuals were collected at specified intervals of 0 (serving as the control), 6, 24, and 96 h post-stress exposure. All samples were placed in tubes and frozen in liquid nitrogen overnight.

### 2.3. Total RNA Extraction and Quality Analysis

Total RNA was extracted by using a Trizol reagent kit (Invitrogen, Carlsbad, CA, USA) according to the manufacturer’s instructions. To eliminate residual DNA, the extracted total RNA was incubated with RNase-free DNase I (Takara Bio, Shiga, Japan) at 37 °C for 30 min. The concentration of RNA was assessed using a NanoDrop 2000 spectrophotometer (Thermo Scientific, Waltham, MA, USA), while agarose gel electrophoresis was utilized to determine the integrity of the RNA samples.

### 2.4. Small RNA Library Construction and Sequencing

Polyacrylamide gel electrophoresis (PAGE) was used to enrich RNA molecules falling within the 18–30 nucleotide (nt) size range. Following this, adapters were introduced and ligated to the RNA molecules. The ligated RNA products were then subjected to reverse transcription via PCR amplification. PCR products ranging from 140–160 bp in length were subsequently enriched to construct a cDNA library, which was sequenced on an Illumina Novaseq 6000 platform at Gene Denovo Biotechnology Co. (Guangzhou, China).

### 2.5. Bioinformatics Analysis of miRNA Transcriptome

Original data obtained from Illumina sequencing were preprocessed using fastp v.0.23.4 [29] to eliminate low-quality reads, adaptors and other contaminative sequences. Filtered reads were subjected to BLAST v.2.13.0 searches against the GeneBank and Rfam databases [30] to remove non-coding RNA sequences (including tRNAs, scRNAs, snoRNAs, snRNAs and rRNAs). Reads mapped to exons, introns and repeat sequences were also removed by aligning to the reference genome of *P. monodon*. The remaining reads were searched against the miRBase database [31] to identify known miRNAs. For unannotated sequences, a search was conducted using miRDeep2 v.0.1.3 [32] to predict novel miRNA candidates.

### 2.6. Expressed Analysis and Target Prediction of miRNA

Transcripts per million (TPM) were used to investigate the expression levels of miRNA. Differentially expressed miRNAs (DEMs) between the control group and experimental group were detected using the DESeq2 package [33] in R. In this study, the threshold for defining differentially expressed miRNAs was set as |log2 fold change| ≥ 1 and *p* value < 0.05. After obtaining the files of differentially expressed miRNAs, trend analysis was performed using the OmicShare tool [34] with default parameters.

The target genes of miRNA were predicted using a variety of software tools, including miRanda v.3.3a [35], RNAhybrid v.2.1.2 [36] and TargetScan v.7.0 [37]. We employed the default parameters of TargetScan to seek miRNA seed matches (2–8 nt sequences starting from the 5′ end of the miRNA) in 3′-UTR sequences. Additionally, miRanda and RNAhybrid were utilized to align entire miRNA sequences. The parameters of miRanda were set to a score >140 and free energy <−20 kcal/mol, respectively. The RNAhybrid software was run using the following parameters: free energy threshold <−20 kcal/mol and helix constraint from 2 to 8. The final set of predicted target genes for DEMs was determined by identifying the overlap between the results obtained from all the prediction tools.

### 2.7. Enrichment Analysis of Target Genes

To gain a better understanding of the biological activities regulated by DEMs enriched in trend modules, Gene Ontology (GO) [38] and KEGG pathway [39] enrichment analyses of target genes were conducted. Based on hypergeometric tests and FDR correction, terms or pathways exhibiting an FDR less than 0.05 were defined as significantly enriched. We employed the OmicShare tool [34] to select and visualize the top 20 results.

### 2.8. Validation of DEMs and Statistical Analysis

To assess the reliability of miRNA expression profiles from high-throughput sequencing, nine random DEMs were selected for the validation of their expression. Then, stem-loop qRT-PCR was conducted. Primers were designed and are listed in Supplementary Table S1. To normalize the miRNA expression levels, U6 snRNA was chosen to be an internal control. Further qPCR experiments were carried out in a LightCycler^®^ 480 II Real-time PCR Instrument (Roche, Basel, Switzerland) using SYBR Green Master Mix (Qiagen, Hilden, Germany) following the manufacturer’s recommendations. Each 25 µL PCR reaction volume was carried out with 12.5 µL PCR mastermix, 2 µL primers, 5.5 µL distilled water, and 5 µL of cDNA template. The amplification protocol consisted of an initial denaturation at 95 °C for 10 s, followed by 40 cycles at 95 °C for 15 s, 60 °C for 30 s, and 72 °C for 30 s. The relative expression of each DEM was calculated using the 2^−ΔΔCt^ method. Finally, the results were statistically analyzed using a one-way ANOVA with SPSS v.23.0 (IBM, Armonk, NY, USA).

## 3. Results

### 3.1. Identification and Classification of miRNAs

**Figure 1:**
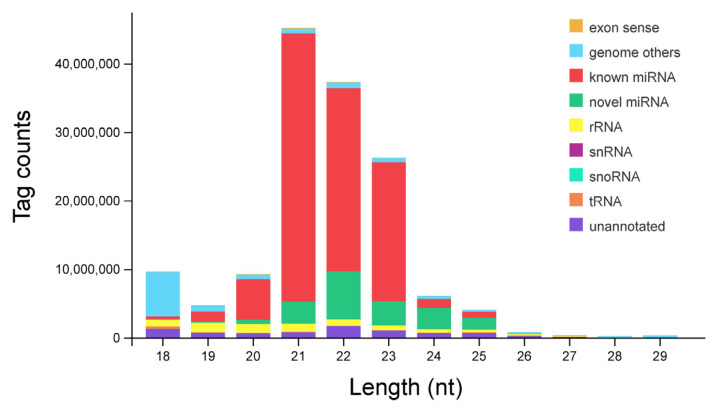
Length distribution of small RNA sequences.

Twelve separate small RNA libraries were constructed from the hepatopancreas of *P. monodon*. Based on high-throughput sequencing, 159,919,915 raw reads were generated. After removing the low-quality and contaminated reads, 145,241,136 (90.82%) clean reads were acquired from all libraries. The sequence length distribution of small RNAs ranged from 18 nt to 29 nt, with 21 nt small RNAs being the most abundant, followed by 22 nt and 23 nt (Figure 1).

For small RNA annotation, the clean reads were aligned to the genome and various databases. A total of 125,553,226 (86.44%) reads were mapped to the genome sequences. Then, small RNAs were annotated and classified into rRNAs, snRNAs, tRNAs, snoRNAs, miRNAs, and unannotated RNAs after being compared with the Rfam and GenBank databases (Figure 1). The number of miRNAs detected in each sample ranged from 479 to 605, with no significant differences (Figure S1). Finally, 684 miRNAs were identified from hepatopancreas tissues in total, including 488 known miRNAs and 196 novel miRNAs (Table S2).

### 3.2. Principal Component Analysis

**Figure 2:**
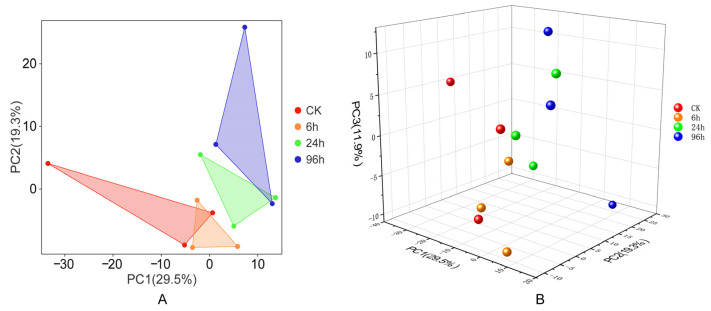
Relationships between samples according to principal component analysis. Groups subjected to different treatments are represented by different colors. (**A**) Visualization of 2D PCA results. (**B**) Visualization of 3D PCA results.

In order to evaluate the relationships between all samples, a principal component analysis was conducted. Based on the two-dimensional results, the groups exhibit some overlap and are not completely separated from each other (Figure 2A). It seems that the 6 h group (6 h) is close to the control group (CK), while the 24 h and 96 h groups are similar. The same results can also be found in Figure 2B, which illustrates the relationships among samples in a three-dimensional representation. As the duration of low-salinity stress increases, the expression of miRNAs exhibits different patterns.

### 3.3. Expression Profiles of Differentially Expressed miRNA

**Figure 3:**
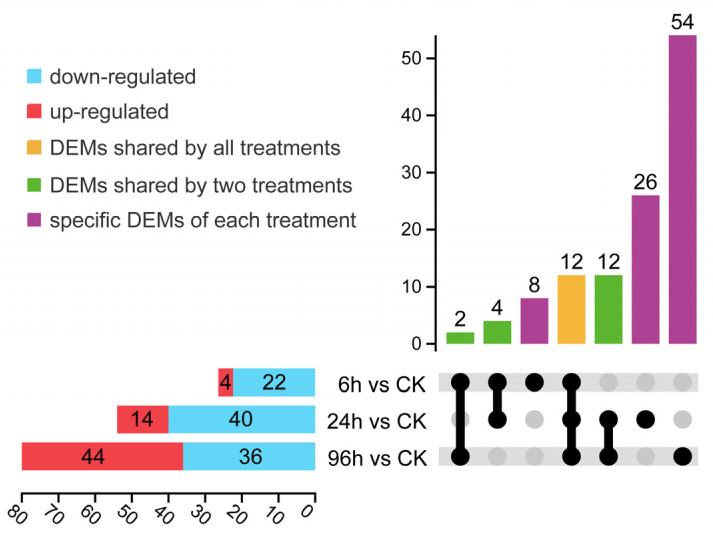
UpSet diagrams based on DEMs at different stages of moderately low salinity treatment.

Compared to the controls, miRNAs with a fold change greater than 2 and a *p* value less than 0.05, were identified as differentially expressed (DEM). Upon exposure to moderately low salinity treatments for 6 h, 24 h, and 96 h, there were 4, 14, and 44 miRNAs that showed up-regulated expression, respectively, while 22, 40, and 36 miRNAs demonstrated down-regulated expression (Figure 3, Table S3). An UpSet diagram analysis of the DEMs revealed that various miRNAs responded to different low-salinity conditions, with a total of 12 miRNAs being differentially expressed across all stages of treatment (Figure 3).

### 3.4. Trend Analysis of Differentially Expressed miRNA

**Figure 4:**
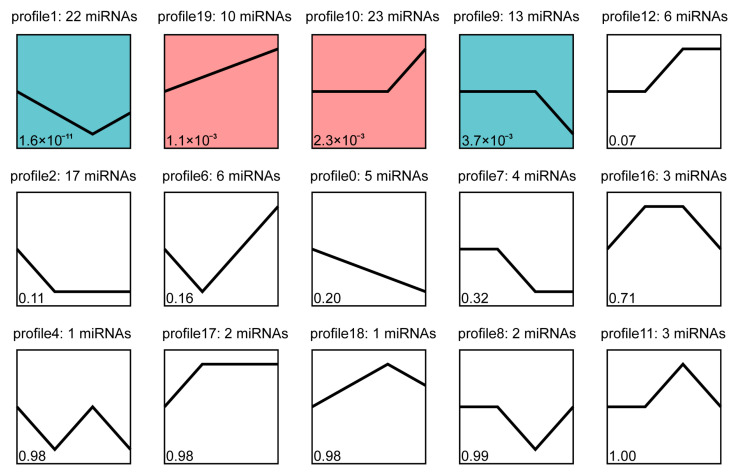
Schematic diagram of miRNA expression trends between treatments. The trend ID and the number of genes it contains are at the top. The colored trend blocks indicate a trend of significant enrichment, and different colors indicate different trends.

To explore the potential role of miRNAs in *P. monodon* under low-salinity stress, we performed an analysis of the expression trends of DEMs. After excluding the profiles that did not enrich any miRNAs, fifteen distinct trend profiles were obtained (Figure 4). Based on *p* values, the DEMs were most enriched in four profiles, including two descending and two rising trend modules. Distinct expression patterns were observed across four significant expression profiles. In profile 19, miRNAs were up-regulated consistently. In profiles 10 and 9, most miRNAs maintained their original expression levels within 24 h and were up-regulated or down-regulated from 24 h to 96 h. In contrast, expression levels of miRNAs in profile 1 were down-regulated over 0–24 h, while being up-regulated from 24 h to 96 h. It is commonly recognized that miRNAs play essential regulatory roles by targeting mRNA for cleavage or translational repression. Consequently, DEMs enriched in those four trend modules (profile 1, profile 9, profile 19, profile 10) were used for further analysis.

### 3.5. Prediction of miRNA Targets and Functional Enrichment Analysis

**Figure 5:**
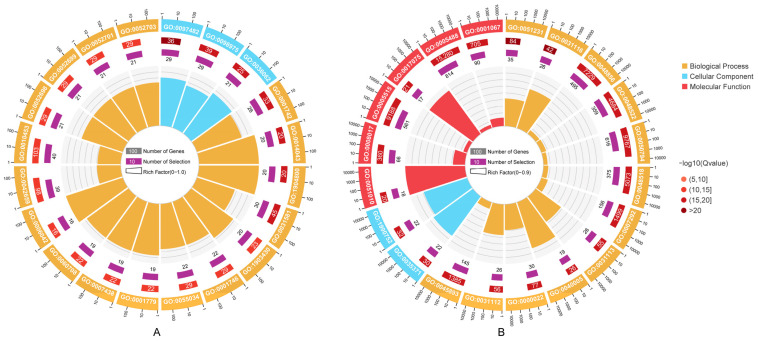
GO enrichment analysis of target genes. (**A**) Genes regulated by miRNA enriched in rising trend modules. (**B**) Genes regulated by miRNA enriched in descending trend modules.

Based on the published genome of *P. monodon*, we predicted putative targets of the DEMs by using multiple tools. For all 118 DEMs, a total of 4907 transcripts were identified as targets, indicating that a single miRNA regulates multiple target genes. Subsequently, the target genes of DEMs enriched in four significant trend modules were subjected to GO enrichment analysis. The top 20 GO terms with the lowest Q values were selected as significantly enriched functional categories (Figure 5). Results of the GO analysis demonstrated that there are significant differences in the functions of target genes regulated by DEMs in rising trend modules (profile 10, profile 19) and descending modules (profile 1, profile 9). For up-regulated DEMs, pathways involved in development (GO:0055034, GO:0014043, GO:0031581, GO:1903430, GO:0007438), differentiation (GO:0001742, GO:0001779, GO:0060708, GO:0001746, GO:1904800), biosynthesis (GO:0006042, GO:0052699, GO:0052703) and metabolism (GO:0052698, GO:0052701) were enriched (Figure 5A). In terms of down-regulated DEMs, top terms were related to microtubule (GO:0031116, GO:0035371, GO:0051010, GO:0008017, GO:1990752), binding (GO:0005515, GO:0017075, GO:0005488, GO:0001067) and regulation processes (GO:0031116, GO:0048522, GO:0048518, GO:0031113, GO:0031112, GO:0045893, GO:0001067) (Figure 5B).

**Figure 6:**
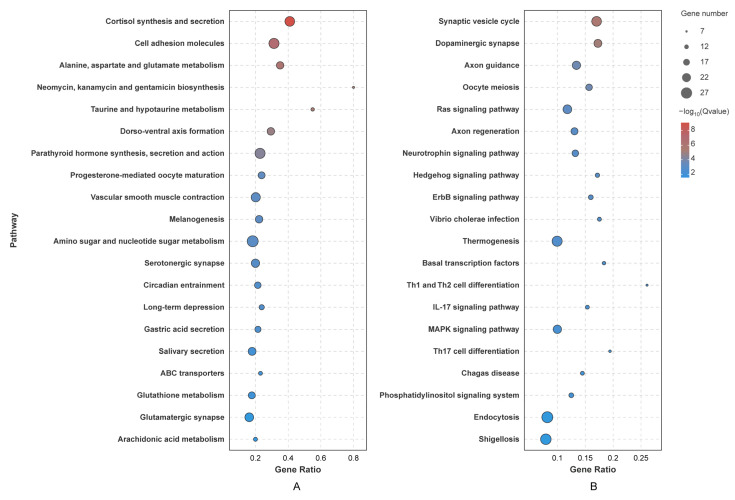
KEGG pathway analysis of target genes. (**A**) Genes regulated by miRNA enriched in rising trend modules. (**B**) Genes regulated by miRNA enriched in descending trend modules.

Similar to the GO analysis, a KEGG pathway analysis was also performed on target genes regulated by DEMs in four significant trend modules. Similar to the GO enrichment analysis, the top 20 significantly enriched KEGG pathways were selected based on Q values. The results showed that target genes could be classified into several different categories, including organismal systems, cellular processes, environmental information processing and metabolism. However, significant differences were also found among pathways associated with different modules (Figure 6). Genes targeted by up-regulated DEMs were most concentrated on metabolism (alanine, aspartate and glutamate metabolism, neomycin, kanamycin and gentamicin biosynthesis, taurine and hypotaurine metabolism, amino sugar and nucleotide sugar metabolism, glutathione metabolism, arachidonic acid metabolism), the endocrine system (cortisol synthesis and secretion, parathyroid hormone synthesis, secretion and action, progesterone-mediated oocyte maturation, melanogenesis), the digestive system (gastric acid secretion, salivary secretion) and development (dorso-ventral axis formation). For down-regulated DEMs, target genes were associated with the nervous system (synaptic vesicle cycle, dopaminergic synapse, axon guidance, axon regeneration, neurotrophin signaling pathway, hedgehog signaling pathway), the immune system (Th1 and Th2 cell differentiation, IL-17 signaling pathway, Th17 cell differentiation), signal transduction (ras signaling pathway, MAPK signaling pathway, phosphatidylinositol signaling system) and environmental adaptation (thermogenesis, endocytosis).

**Figure 7:**
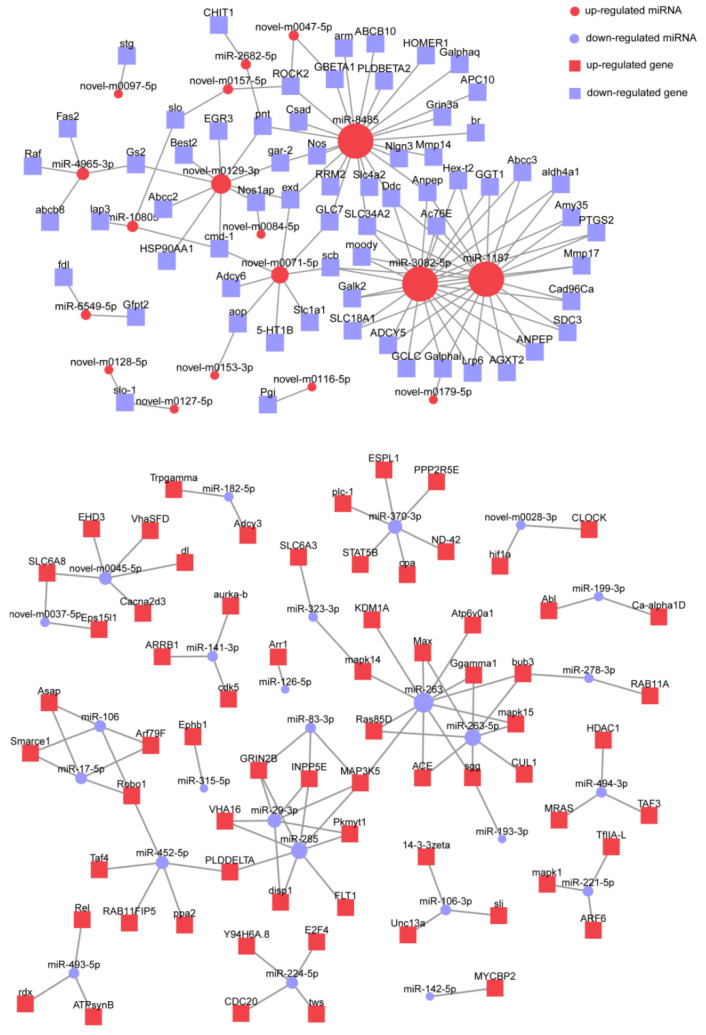
Interactions between DEMs and target genes enriched in KEGG pathway.

Furthermore, the interactions between miRNAs and their target genes enriched in the top 20 KEGG pathways were predicted. The results showed that miRNAs with different expression trends also have different regulatory patterns (Figure 7). The down-regulated target genes are primarily controlled by several miRNAs, such as miR-8485, miR-3082, miR-1187, novel-m0129 and novel-m0071. On the contrary, genes targeted by down-regulated miRNAs are relatively dispersed. More down-regulated miRNAs participated in the top 20 KEGG pathways. It is noteworthy that some genes are regulated by three or more miRNAs, which are listed in Table 1. Functional analysis shows that these common target genes are primarily involved in processes such as metabolism, signal transduction, development and differentiation, and cytoskeleton regulation.

### 3.6. Stem-Loop qPCR Validation of Differentially Expressed miRNA

**Figure 8:**
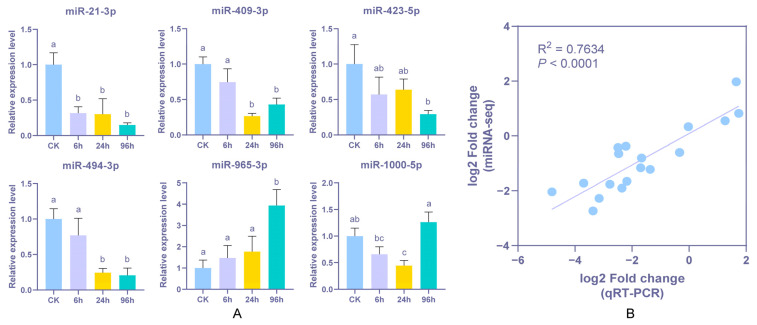
Expression levels of six miRNAs. Bars with different letters indicate significant differences (**A**). Comparison of relative fold change obtained from qPCR analysis and miRNA-seq data (**B**).

To verify the reliability of the high-throughput sequencing and bioinformatics analysis, six DEMs were randomly selected and subjected to stem-loop qPCR validation (Figure 8). The results revealed that the expression levels of these DEMs were consistent with the results of miRNA sequencing. The correlation coefficient of regression analysis between these two approaches was calculated to be 0.7634 (*p* < 0.0001), indicating that the miRNA expression profiling of sequencing data can reflect reliable expression levels in *P. monodon* hepatopancreas tissue under low-salinity treatment. Further analysis predicted 406 target genes regulated by the 6 DEMs, which are primarily associated with nervous system function, signal transduction, development, and regeneration, suggesting that miRNAs play a critical role in low salinity adaptation (Table S4).

## 4. Discussion

As an environmental factor, salinity significantly impacted various parameters of the black tiger shrimp, including growth, survival and gene expression. In recent years, heavy rainfall caused by global climate change has frequently been observed in southern China, resulting in a decrease in water salinity from 30 ppt to below 20 ppt, which has severely affected the development of the *P. monodon* aquaculture industry [11]. Thus, there is an urgent need to understand how low salinity affects shrimp. Over the past few decades, a large number of miRNAs have been identified and proven to serve as key players in a robust adaptive response against stress in aquatic animals [21,22,23]. However, there are relatively few reports on the role miRNAs play in salinity response in crustaceans, especially *P. monodon*. In a previous study, we predicted the molecular mechanisms of *P. monodon* under severe low-salinity stress (3 ppt) [24]. Nevertheless, studies have indicated that varying low-salinity gradients differentially affect the physiological, biochemical, and gene expression profiles of *P. monodon* [6]. This suggests that distinct regulatory networks are activated in *P. monodon* to cope with different low-salinity conditions. Therefore, to better understand the molecular mechanisms underlying its low-salinity adaptation, research should focus not only on extreme low-salinity stress but also on moderately low salinity gradients. Based on small RNA transcriptome sequencing and bioinformatics analyses, a total of 684 microRNAs were identified from the hepatopancreas, including 481 known miRNAs, 196 novel miRNAs and 7 conserved miRNAs. The length of the miRNAs was mainly concentrated between 21 and 23 nt, which was consistent with other observations in *P. monodon* [40]. The miRNAs identified in this study lay the groundwork for future investigations into shrimp miRNAs and will help to fill the knowledge gap regarding their function in shrimp stress response.

To identify the miRNAs involved in the response to low-salinity exposure, miRNA expression levels at 6 h, 24 h and 96 h were compared with those in controls. A total of 118 microRNAs were differentially expressed, implying their potential roles in stress response. Compared with our previous work, the number of DEMs was lower. The reason for this might be that moderately low salinity has a minor impact on *P. monodon* and there is no need for a large number of DEMs to cope with stress. Similar results were also found in a previous study, which indicated that a salinity of 10 ppt had less effect on growth and gene expression compared to salinities of 5 ppt and 2.5 ppt [6]. Moreover, to investigate the expression patterns of candidate miRNAs in response to low salinity, DEMs were subjected to trend analysis. Finally, 68 DEMs with either rising or descending trends were significantly enriched and selected for subsequent analysis. As key endogenous mediators of RNA interference (RNAi), miRNAs are capable of modulating various biological processes in organisms, such as cell differentiation, development, metabolism, and apoptosis [41]. The expression patterns of DEMs suggested that a series of target genes were repressed or activated in *P. monodon* under low-salinity stress. Similarly, in a transcriptome study, it was found that low salinity induced the differential expression of genes, including 1140 up-regulated genes and 1531 down-regulated genes [42].

The target genes of DEMs were predicted using bioinformatic methods to characterize the function of miRNAs in shrimp stress response to low-salinity exposure. However, the prediction of target genes varies among software tools. Referring to published works in *L. vannamei* [20] and *Fenneropenaeus chinensis* [22], we employed multiple software tools to obtain target genes in the present study. A total of 4907 transcripts were identified as the targets of 118 DEMs, indicating that a single miRNA could regulate numerous target genes. Furthermore, enrichment analysis was conducted to elucidate the biological function of these predicated target genes. According to GO analysis, genes targeted by up-regulated DEMs were highly enriched in terms of development, differentiation, biosynthesis and metabolism. This might suggest that the growth of *P. monodon* was restricted by low salinity, which is consistent with previous studies on shrimp [6,11]. On the contrary, genes controlled by down-regulated DEMs were concentrated on microtubule, binding and regulation processes, indicating that a series of stress-related responses were activated to maintain cellular homeostasis [24]. Similarly, in the miRNA transcriptomes of *Apostichopus japonicus*, the salinity adaptation process was related to ion channels, transporters, and environmental signals, indicating the regulatory roles of miRNAs [43].

KEGG pathway analysis of potential target genes provides a powerful approach to investigate the molecular mechanisms of *P. monodon* under low-salinity stress conditions. In this study, target genes involved in amino acid metabolism, amino sugar metabolism and arachidonic acid metabolism were governed by up-regulated miRNAs. Amino acid metabolism is critical for physiological homeostasis [44]. Previous studies have demonstrated that stress significantly disrupts amino acid metabolic pathways in *L. vannamei*, resulting in a decrease in most amino acid concentrations [45]. Amino sugar metabolism, particularly chitin synthesis, is essential for crustacean exoskeletal integrity, which plays a potential role in growth [46]. As an important fatty acid, arachidonic acid could enhance the antioxidant and immune capacity of fish, resulting in improved growth [47]. Given that miRNAs function to silence mRNA expression, we can hypothesize that low-salinity stress suppresses metabolic processes in *P. monodon,* potentially further impacting its growth. Moreover, genes related to the digestive and endocrine systems are also managed by up-regulated miRNAs. Salivary and gastric acid are responsible for food digestion and mineral absorption. A decrease in salivary and gastric acid secretion means that low salinity affected the feeding behavior of shrimp, which could be a contributing factor to the slow growth observed in shrimp under low-salinity conditions [6]. Cortisol and parathyroid hormones, which belong to the endocrine system, play crucial roles in dealing with harsh environments [48,49]. In our study, changes in these two hormones suggested that energy metabolism and immune response were affected by stress. Based on the above results, it can be inferred that low salinity has impacts on a series of biological processes related to metabolism, digestion and hormone synthesis, ultimately causing retarded growth in *P. monodon*.

Low salinity can cause damage to the hepatopancreas and compromise the immune system, leading to outbreaks of disease and death in shrimp. When *L. vannamei* were exposed to low-salinity stress, the levels of *Vibrio* infection increased [4]. In this research, three pathways involved in pathogen infection were also significantly enriched, indicating the risk of disease outbreak. For crustaceans, the innate immune response is crucial for defense against microbial infections. Being closely associated with innate immune cells, endocytosis is important for maintaining normal cellular function by removing foreign substances and protecting the host from pathogen/virus attacks. A comparative proteome analysis of the hepatopancreas in *L. vannamei* revealed a significant up-regulation of the protein associated with endocytosis under long-term low-salinity stress [50]. Similar results were detected in this study, implying the potential role of endocytosis in shrimp innate immunity. It is commonly acknowledged that invertebrates, including crustaceans, do not possess an acquired immune system. Recently, increasing evidence has suggested that a specific acquired immunity may exist in invertebrates, which is called ‘immune priming’ [51]. After challenge with WSSV, pathways related to acquired immunity, such as Th1 and Th2 cell differentiation, the IL-17 signaling pathway and Th17 cell differentiation were detected in Pacific white shrimp [52]. Additionally, another study implied that thermogenesis may play a key role in the interactions between shrimp and pathogens across tissues [53]. Interestingly, these immune-related pathways were also found in this research, reflecting immune priming in *P. monodon*. These pathway changes indicate that low salinity increased disease susceptibility, triggering various immune responses in the hepatopancreas of *P. monodon*.

Although *P. monodon* has euryhalinity, its survival depends on its ability to sense and respond to salinity changes. As a key mediator of the stress response, the nervous system gathers information from sensory organs, processes it, and then coordinates actions. It was reported that the nervous system in crustaceans has the capability to regulate numerous physiological processes, thereby maintaining the organism’s homeostasis under stressful conditions [54]. From the genome of *L. vannamei*, genes related to neural development are notably enriched for environment adaptation [55]. Our results showed that pathways related to synapse and axon were enriched under low salinity, which was consistent with a previous study [24]. It can be inferred that the nervous system is vital in adapting to salinity changes in *P. monodon*. Signal transduction serves as a bridge that connects sensing and responding. The renin-angiotensin system (RAS) is a hormone-based system that participates in osmoregulation and salinity adaptation by regulating the water balance and blood pressure of teleost fish [56]. A comparative analysis of Pacific white shrimp showed that the RAS signaling pathway was up-regulated under multiple environmental stressors [57]. As receptors for growth factors, the ErbB family mediate a variety of cellular responses. In aquatic animals, the ErbB signal is involved in development, homeostasis and pathologies [58,59]. Similar to ErbB, the mitogen-activated protein kinase (MAPK) pathway is a multifunctional signaling pathway which can modulate diverse cellular function, including growth, differentiation and responses to external stress. It has been demonstrated that genes linked to the MAPK signaling pathway are up-regulated in shrimp in response to low-salinity stress [14]. As a vital component of cell membranes, phosphatidylinositol is essential for signal transduction in response to osmotic stress. In *L. vannamei*, dietary myo-inositol can regulate osmotic pressure through the phosphatidylinositol signaling pathway, ultimately alleviating the damage induced by low-salinity stress [60]. Due to the down-regulation of relevant miRNAs, we can conclude that various signal pathways were activated to cope with acute low-salinity stress.

## 5. Conclusions

This study has demonstrated the regulatory role of miRNAs in black tiger shrimp under moderately low salinity stress (17 ppt). While low salinity impaired growth processes and increased disease vulnerability, miRNAs modulated compensatory pathways to alleviate stress effects, such as signal transduction and immune priming. These findings contrast with prior studies on severe salinity stress, highlighting distinct molecular mechanisms in moderate conditions. The identification of stress-responsive miRNAs provides potential biomarkers for breeding programs aimed at improving salinity tolerance in shrimp aquaculture. Further functional validation of these miRNAs could support the development of resilient shrimp strains, aiding sustainable aquaculture in fluctuating environments.

## Figures and Tables

**Table 1:** Details of common target genes.

Gene Symbol	Description	Related miRNA	Gene Function
Anpep	aminopeptidase N-like	miR-1187, miR-3082-5p, miR-8485	Amino acid metabolism
cmd-1	calmodulin isoform X1	miR-10805, novel-m0071-5p, novel-m0129-3p	Signal transduction
Ddc	aromatic-L-amino-acid decarboxylase-like	miR-1187, miR-3082-5p, miR-8485	Amino acid metabolism
exd	homeobox protein extradenticle-like isoform X1	miR-8485, novel-m0071-5p, novel-m0129-3p	Development and differentiation
Galphai	guanine nucleotide-binding protein G(i) subunit alpha isoform X1	miR-1187, miR-3082-5p, novel-m0179-5p	Lipid metabolism
pnt	ETS-like protein pointed isoform X1	miR-2682-5p, miR-8485, novel-m0129-3p	Development and differentiation
ROCK	rho-associated protein kinase 1-like	miR-8485, novel-m0047-5p, novel-m0157-5p	Regulation of cytoskeleton
scb	integrin alpha-7-like	miR-1187, miR-3082-5p, novel-m0071-5p	Regulation of cytoskeleton
SLC34A2	sodium-dependent phosphate transport protein 2B-like	miR-1187, miR-3082-5p, miR-8485	Phosphate digestive absorption
MAP3K5	mitogen-activated protein kinase kinase kinase 15-like isoform X1	miR-263, miR-285, miR-29-3p, miR-83-3p	Signal transduction
bub3	mitotic checkpoint protein BUB3-like isoform X1	miR-263-5p, miR-263, miR-278-3p	Pathogen infection
GRIN2B	glutamate receptor ionotropic, NMDA 2B-like isoform X1	miR-285, miR-29-3p, miR-83-3p	Synapse maturation and plasticity
INPP5E	phosphatidylinositol polyphosphate 5-phosphatase type IV-like isoform X1	miR-285, miR-29-3p, miR-83-3p	Signal transduction
Robo1	protein sax-3-like isoform X1	miR-106, miR-17-5p, miR-452-5p	Axon guidance and regeneration

## Data Availability

Data will be made available on request.

## Supplementary Materials

The following supporting information can be downloaded at: https://www.mdpi.com/article/10.3390/biology14040440/s1, Figure S1: Number of miRNAs detected in each sample; Table S1: Primers used for miRNA expression validation; Table S2: Detected miRNAs and their expression levels; Table S3: Expression patterns of differentially expressed miRNAs across experimental groups; Table S4: Target genes regulated by six differentially expressed miRNAs.
