# Supplementary figures and images for: Insights into the Regulatory Role of MicroRNAs in Penaeus monodon Under Moderately Low Salinity Stress
Source: Biology (Basel). 2025 Apr 18;14(4):440. doi: 10.3390/biology14040440 (PMC12024918; doi:10.3390/biology14040440)

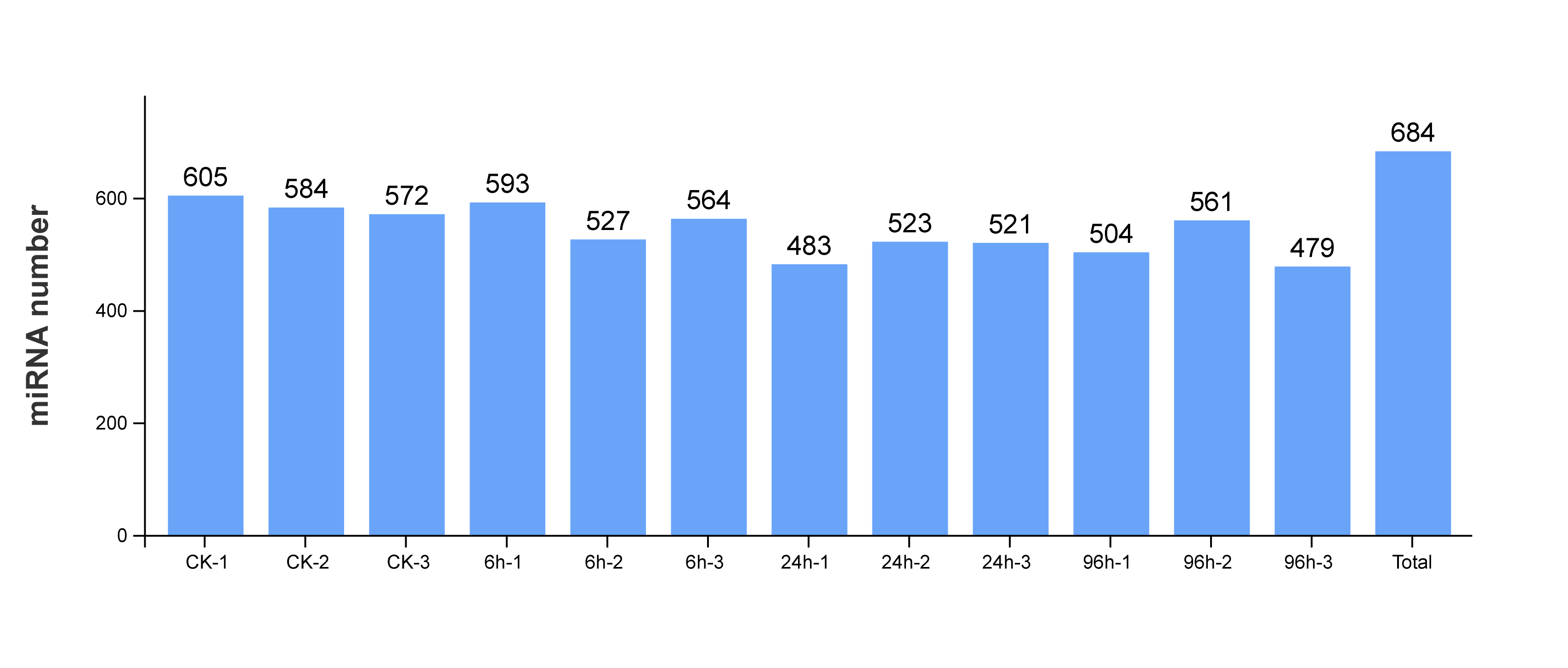

Supplement: Supplementary file 1 [file biology-14-00440-s001.zip › Fig. S1.tif]
